# Association of serum lipids and severity of epithelial ovarian cancer: an observational cohort study of 349 Chinese patients

**DOI:** 10.7555/JBR.32.20170096

**Published:** 2018-02-23

**Authors:** Yi Zhang, Jing Wu, Junya Liang, Xing Huang, Lei Xia, Dawei Ma, Xinyu Xu, Pingping Wu

**Affiliations:** 1. Department of Pathology, Jiangsu Cancer Hospital, Jiangsu Institute of Cancer Research, the Affiliated Cancer Hospital of Nanjing Medical University, Nanjing, Jiangsu 210009, China; 2. Department of Radiation Oncology, Jiangsu Cancer Hospital, Jiangsu Institute of Cancer Research, the Affiliated Cancer Hospital of Nanjing Medical University, Nanjing, Jiangsu 210009, China; 3. The School of Public Health, Nanjing Medical University, Nanjing, Jiangsu 211166, China; 4. Department of Oncology, Jiangsu Cancer Hospital, Jiangsu Institute of Cancer Research, the Affiliated Cancer Hospital of Nanjing Medical University, Nanjing, Jiangsu 210009, China.

**Keywords:** triglycerides, epithelial ovarian cancer, high density lipoproteins, dyslipidemia, clinical data statistics

## Abstract

While obesity and fat intake have been associated with the risk and prognosis of epithelial ovarian cancer, the association between the lipid levels and epithelial ovarian cancer phenotype remains controversial. We conducted a retrospective study of 349 epithelial ovarian cancer patients who received treatment at Jiangsu Cancer Hospital, China between 2011 and 2017. We analyzed age at diagnosis, blood pressure, plasma glucose content, body mass index (BMI), lipid levels and clinical parameters. Severity of epithelial ovarian cancer was classified according to the International Federation of Gynecology and Obstetrics (FIGO) grading system. Univariate analysis of the clinical factors according to the severity of epithelial ovarian cancer was followed by logistic regression analysis to identify clinical factors significantly associated with epithelial ovarian cancer severity. Univariate analysis indicated that age, BMI, triglyceride (TG), and high density lipoproteins (HDL) differed significantly among different stages of epithelial ovarian cancer (*P*<0.05). In the logistic regression model, elevated TG (OR: 1.883; 95% CI= 1.207–2.937), and low HDL (OR: 0.497; 95% CI= 0.298–0.829) levels were significantly associated with the high severity epithelial ovarian cancer. Our data indicate that high TG and low HDL levels correlate with a high severity of epithelial ovarian cancer. These data provide important insight into the potential relationship between the lipid pathway and epithelial ovarian cancer phenotype and development.

## Introduction

Ovarian cancer is one of the most lethal gynecological malignancies worldwide, with an estimated 21,290 new diagnoses of ovarian cancer and 14,180 deaths anticipated for 2015 in the United States^[1]^. Epithelial ovarian cancer (EOC) accounts for 90% of ovarian cancer cases, with serous cancer accounting for the most common histology type^[2]^. Most women present with advanced-stage disease at the time of diagnosis, resulting in elevated mortality rates^[3]^. However, because of the multiple causes, scientists have not reached a consensus concerning the prevalence of EOC. Therefore, a marker that could be associated with tumor progression is necessary^[4]^.

The risk factors of EOC include age at diagnosis, family history of EOC, infertility treatment and assisted fertilization, obesity and metabolic syndrome^[5]^. More recently, in terms of the incidence and outcome of EOC patients, attention is being paid to metabolic conditions such as obesity, fatty acids and diabetes mellitus^[6]^. Increasing evidence has suggested that obesity is a significant risk factor for EOC and is associated with worse outcomes for this disease^[7]^. Beavis *et al.*^[8]^ reported that individuals with obesity are at increased risk of ovarian cancer, and Erondu *et al.*^[9]^ also observed that the level of body mass index (BMI) was significantly associated with symptoms of EOC patients. Lees *et al.*^[10]^ also clarified that diabetes is associated with gynecologic cancer.

Therefore, the extensive literature has reported on the relationship between metabolic markers of obesity including elevated blood glucose, triglyceride (TG) and total cholesterol (TC) levels, and ovarian cancer^[11]^. For instance, high cholesterol and low-density lipoprotein (LDL) levels are one of the most important and influential factors in the etiology of different types of cancers, including ovarian cancer^[12]^. Moreover, collective data have supported that LDL could be a significant predictor of clinical outcome in advanced-stage epithelial cancer^[13]^. In addition, a previous study clarified that elevated TG was linked with cancer risk in Chinese patients with EOC^[14]^.

However, limited studies are available concerning the relationship between obesity, as well as lipid levels, and EOC cancer stage in Chinese individuals. Thus, we designed this retrospective study of EOC to explore the link between the levels of serum lipid components and cancer stage of EOC patients, as a possible underlying mechanism of the association between obesity and cancer.

## Patients and methods

### The study population

The study population was collected from patients undergoing treatment at Jiangsu Cancer Hospital, which is the largest cancer hospital in Jiangsu Province. Between October 2011 and June 2017, 349 patients who were histologically diagnosed with ovarian serous cancer were recruited as subjects. All patients in the samples were collected before surgery, radiotherapy and chemotherapy and were excluded if they had a history of abnormal coronary heart disease, stroke, thyroid disease, liver and kidney disease, polycystic ovary syndrome and other related to lipid metabolism diseases. The study was approved by the ethics committee of Nanjing Medical University. Informed consent was not required because of the retrospective nature of the study.

### Data collection procedures

The clinical data were reviewed from medical records, including age, blood pressure, level of glucose, BMI level, lipid levels, and clinical parameters such as volume of ascetic fluid and pathologic stage. Lipid levels were recorded from the patients’ first visit to the hospital prior to any intervention.

Disease severity was classified according to the International Federation of Gynecology and Obstetrics (FIGO) grading system. Considering the therapeutic method of EOC for different stages, low severity was defined as FIGO Ⅰ–Ⅱ stage; high severity was defined as FIGO Ⅲ–Ⅳ stage.

### Statistical analysis

Statistical analysis was performed using IBM SPSS version 20.0 (IBM Corp., Armonk, NY, USA). The demographic and baseline variables related to cancer staging were tested by chi-square test. The characteristics were expressed as means (standard deviation) for continuous variables. The significance of the mean differences was tested by Student’s *t*-test. For univariate analysis, the authors used 0.15 as the criterion for variables to be selected for multivariate analysis. Unadjusted and adjusted logistic regression analysis was performed to determine the factors that can affect the severity staging. Then the logistic regression analysis model using a stepwise regression method was made to confirm the parameters associated with EOC severity. A *P*-value<0.05 was significant.

## Results

**Fig.1:**
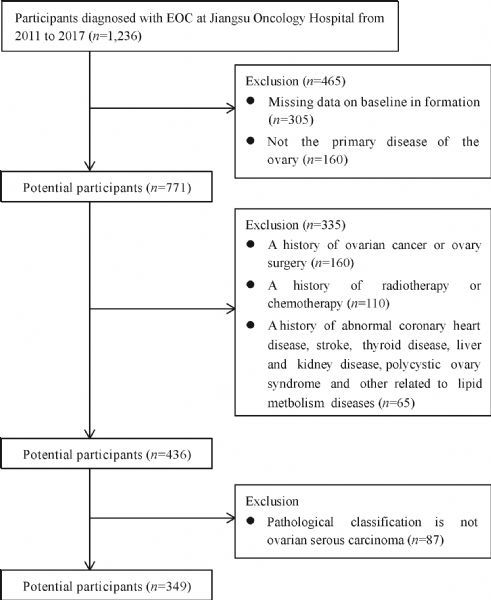
The study flowchart.

The study flowchart illustrating the selection of the study subjects is shown in ***Fig. 1***. After the eligibility review, 349 EOC patients were enrolled in the analysis, and their characteristics related to cancer staging are presented in ***Table 1***.

**Tab.1:** Demographics and clinical characteristics of the study population (*n* = 349)

Characteristics	Low severity (*n* = 140) [*n* (%)]	High severity (*n* = 209) [*n* (%)]	*P*-value
Age at diagnosis (years)			
≤50	60(42.8)	109(57.4)	0.147*
BMI (kg/m^2^)			
≤25	97(69.3)	115(55)	0.007*
Smoking history			
Yes	29(20.7)	53(25.3)	0.316
Family history of ovarian cancer			
Yes	43(30.7)	77(36.8)	0.237
Oral contraceptive use			
Yes	122(87.1)	174(83.3)	0.321
Ascites			
Yes	95(67.9)	139(66.5)	0.293
Tumor location			
Unilateral	106(75.7)	157(75.1)	0.299
Bilateral	34(24.3)	52(24.9)	
Lymph node metastasis			
Yes	15(10.7)	100(47.8)	0.212
Distant metastasis			
Yes	15(10.7)	19(9)	0.616

*P*-value from chi-square test. * denotes *P*<0.15. BMI: body mass index.

***Table 2*** describes the clinical characteristics of all study subjects. The high severity patients were significantly older, and had higher BMI, TG levels and lower HDL levels. The EOC severity varied significantly by age at cancer diagnosis, BMI, TG, and HDL levels (*P*<0.15).

**Tab.2:** Patient demographic and clinical characteristics stratified by severity of EOC

Group	Low severity	High severity	*t*	*P*-value
N	140	209	/	/
Age (years)	53.44±9.58	55.56±9.74	κ-2.004	0.046*
DBP (mmHg)	73.93±9.49	73.70±9.22	0.226	0.822
SBP (mmHg)	120.01±14.93	120.30±15.48	κ-0.177	0.860
BMI (kg/m^2^)	23.61±3.52	24.40±2.92	κ-2.277	0.023*
FBP (mmol/L)	5.30±0.88	5.38±0.91	κ-0.798	0.425
TC (mmol/L)	4.48±0.98	4.64±0.83	κ-1.708	0.089*
TG (mmol/L)	1.28±0.48	1.45±0.56	κ-3.047	0.003*
HDL (mmol/L)	1.34±0.60	1.20±0.32	2.625	0.009*
LDL (mmol/L)	2.90±0.64	2.91±0.62	κ-0.168	0.866

*P*-value from *t*-test. * denotes *P*<0.15. DBP: diastolic blood pressure; SBP: systolic blood pressure; FBP: fasting blood sugar; TC: total cholesterol; TG: triglyceride; HDL: high density lipoproteins; LDL: low-density lipoproteins.

**Tab.3:** Logistic regression models of factors associated with EOC severity using an “Enter” method

Characteristic	Unadjusted OR (95% CI)	*P*-value	Adjusted OR^a^ (95% CI)	*P*-value
Age	1.023 (1.000–1.046)	0.047	1.017 (0.991–1.044)	0.191
DBP	0.997 (0.975–1.021)	0.821	0.998 (0.970–1.028)	0.906
SBP	1.001 (0.987–1.015)	0.859	0.994 (0.975–1.013)	0.527
BMI	1.083 (1.010–1.160)	0.024*	1.073 (0.997–1.155)	0.061
FBP	1.104 (0.866–1.408)	0.425	0.990 (0.762–1.286)	0.941
TC	1.243 (0.967–1.597)	0.089	1.141 (0.869–1.498)	0.342
TG	1.920 (1.231–2.997)	0.004*	1.615 (1.011–2.579)	0.045*
HDL	0.491 (0.297–0.810)	0.005*	0.465 (0.272–0.792)	0.005*
LDL	1.030 (0.731–1.451)	0.866	0.909 (0.631–1.308)	0.607

^a^Adjusted by all variables in the model. *denotes *P*<0.05.

***Table 3*** shows that in the unadjusted and adjusted logistic regression model, high TG levels and low HDL levels were significantly associated with EOC severity (*P*<0.05). Then, the logistic regression analysis model using a stepwise regression method indicated that TG (*P*<0.05) was positively correlated with EOC severity, while HDL (*P*<0.05) was negatively correlated with EOC severity (***Table 4***).

**Tab.4:** Logistic regression models of factors associated with EOC severity using a stepwise regression method

Risk factor	β	*P*-value	OR	95% CI of OR	
				Lower bound	Up bound
TG	0.633	0.005*	1.883	1.207	2.937
HDL	κ-0.699	0.007*	0.497	0.298	0.829

* denotes *P*<0.05.

## Discussion

Ovarian cancer is one of the most lethal gynecological malignancies^[15]^. The exact etiology of ovarian cancer remains unclear, but it has been correlated with obesity and overweight^[16]^. Although numerous investigations have demonstrated altered systemic lipid metabolism in cancer patients, few data has examined an inverse association between serum lipid levels and EOC cancer severity in Chinese patients^[17]^. Our results demonstrate that TG was positively correlated with the severity of EOC, while HDL was negatively correlated with the severity of cancer.

At the beginning of this study, we observed the clinical metabolic characteristics of participants. First, we found the age of high-grade ovarian cancer patients was older, a finding similar to that in a previous epidemiological study^[18]^. Next, the research indicated that the high-grade ovarian cancer group had higher blood pressure and serum glucose. However, we found no statistically significant differences in the blood pressure and serum glucose among the high and low severity ovarian cancer. Presently, accumulating epidemiological literature has demonstrated that metabolic syndrome (MetS) is closely related to the occurrence and development of several cancers^[19]^. The limitations imposed by sample size can be used to explain the phenomenon. When we analyzed the relationship between preoperative BMI and EOC severity, our data suggest that elevated BMI is associated with a highly deteriorated cancer phase. Nevertheless, the tendency lost significance upon multivariate adjustments, a finding that is inconsistent with that in a previous study^[20]^. We think that BMI is not an accurate measure of body fat, regardless of body fat. In addition, it may mislead the body of the lean body.

Previous study reported that cholesterol is included in the lipid bilayer of the cell membrane and this includes the cell membrane of cancer cells^[21]^. We found no statistically significant differences in the total serum cholesterol levels. Many studies have suggested that the formation of lipid rafts in cancer cells facilitate signaling that foster carcinogenic transformation over time^[22]^. Because our study was a retrospective study, the results were limited by the lack of longitudinal data; therefore, we could not assess the effect of patients with high or low cholesterol levels and EOC phenotypes on the time range.

In our study group, high HDL levels showed low-severity ovarian cancer. Many researchers reported that HDL plays a protective role in the tumorigenesis and progression of cancers. Yvan-Charvet *et al.*^[23]^ and Esteve *et al.*^[24]^ revealed the idea that the HDL levels are related to the cytokine levels. On the one hand, increased HDL levels can cause decreased circulating levels of proinflammatory cytokines, such as interleukin 6 (IL-6) and tumor necrosis factor (TNF-α)^[25]^. On the other hand, elevated HDL levels can produce increased levels of anti-inflammatory cytokines, such as IL-10^[26]^. These proinflammatory cytokines have a close relationship with cell proliferation and apoptosis^[27]^. Additionally, it was reported that HDL can reduce the PON-1(paraoxonase) free-radical-scavenging ability by inhibiting the association of PON-1, thus providing some protection for the severity of cancer^[28]^.

A recent study reported the RRs of 1.16 (95% CI: 1.06–1.26) in men and 1.15 (95% CI: 1.05–1.27) in women for total cancer with the top quintile compared with the bottom quintile of TG, suggesting a potential role of TG in cancer development^[29]^. When we analyzed the correlation of TG and EOC severity, our data suggest that elevated TG levels refer to high cancer severity. Similarly, a previous study found a positive association between triglycerides and prostate cancer severity^[30]^. Cancer cells are closely related to lipid molecules, particularly fatty acids, as membrane structures, energy sources and structural regions of related signaling molecules^[31]^. It has been suggested that triglyceride metabolism provides essential fatty acids and can have crucial impacts on the biophysical structure of membranes^[32]^. The high cost of fatty acids in cancer cells can cause the high level of serum TG. Moreover, increasing evidence has suggested that TG may correlate with the insulin status and represent the complex effects on carcinogenesis pathways^[33]^. Insulin increases the production of free insulin-like growth factor-1, which is a critical mitogenic molecule that influences cell growth^[34]^. Meanwhile, an elevated level of insulin induces inflammation, which leads to the induction of cyclooxygenase-2 (COX-2) expression and subsequent prostaglandin (PG) induction, which may directly favor ovarian carcinogenesis^[35]^.

In addition, many *in vitro* studies have shown that melatonin-rich residue particles can cause tumor growth by modulating cell signaling pathways, such as MEK/ERK and PI3K/Akt/mTOR pathways^[36]^. These pathways can have an effect on cell growth and proliferation, apoptosis, cell cycle arrest, and lipid biosynthesis^[37]^, which should be further confirmed in an *in vivo* model.

To our knowledge, few data has examined the relationship between serum lipid levels and EOC severity phenotype in the Chinese population. The hospital from which we collected the information is considered the leading oncology hospital in Jiangsu province. In our study, we found that high TG levels and low HDL levels are correlated with high-severity ovarian cancer. Our ROC analysis revealed that the synergistic effect between TG and HDL may be associated with EOC tumor severity. These two markers may interact with each other and contribute to the pathogenesis of EOC malignancy.

Additionally, the current study has notable limitations. First, our findings are only from a Chinese hospital and do not rule out the possibility of selective bias or sampling error. Second, the lack of a patient's longitudinal history may prevent us from establishing a direct relationship between lipid levels and the severity of EOC. In future studies, a large and randomized population study may ultimately clarify the role of TG and HDL according to EOC severity. In addition, further studies may encompass the mechanisms regarding how TG and HDL affects EOC stage using animal models.

In conclusions, despite limitations in sample size and longitudinal data, this study revealed the possible impact of lipid on EOC phenotype. The potential clinical implication is that EOC severity among Chinese patients was positively associated with elevated TG levels and low HDL levels. Additionally, these lipid levels should be evaluated as part of routine screening and diagnostic procedures.
